# Calcium-induced chloride secretion is decreased by Resveratrol in ileal porcine tissue

**DOI:** 10.1186/s13104-018-3825-4

**Published:** 2018-10-11

**Authors:** Susanne Hoppe, Gerhard Breves, Stefanie Klinger

**Affiliations:** Department of Physiology, University of Veterinary Medicine Hannover, Foundation, Bischofsholer Damm 15, 30173 Hannover, Germany

**Keywords:** CALCIUM, cAMP, Carbachol, Chloride secretion, CFTR, Resveratrol, Short circuit currents, Ussing chamber

## Abstract

**Objective:**

Chloride (Cl^−^) secretion is crucial for intestinal fluid secretion. Therefore, effects of the polyphenol Resveratrol (RSV) on Cl^−^ secretion have been investigated. In a previous study, we observed effects of RSV on forskolin-induced Cl^−^ secretion in the porcine jejunum but not the ileum although RSV itself induced a transepithelial ion current that may represent Cl^−^ secretion in the ileum. The aim of this study was to gain further insights regarding the effects of RSV on characteristics of Cl^−^ secretion in the porcine ileum using the Ussing chamber technique (recording of short circuit currents (I_sc_) as a measure for epithelial net ion transfer).

**Results:**

RSV increased the I_sc_ in the porcine ileum but not in the porcine jejunum as is already known. This increase was absent in a Cl^−^-free buffer system, indicating that RSV indeed induces Cl^−^ secretion. However, the carbachol-induced I_sc_ was significantly inhibited by RSV indicating an inhibition of Ca^2+^-induced Cl^−^ secretion. The cellular basis for these contradictory, segment specific results of RSV on Cl^−^ secretion has to be subjected to further studies. The results also underline, that is difficult to generalize effects of RSV between different intestinal locations, organs, cell culture models or species.

**Electronic supplementary material:**

The online version of this article (10.1186/s13104-018-3825-4) contains supplementary material, which is available to authorized users.

## Introduction

Chloride (Cl^−^) secretion is crucial for intestinal fluid balance since it controls the water transport into the gut lumen and is thus involved in the development of secretory diarrhea. Reduced Cl^−^ secretion decreases water movement into the gut lumen and can thus result in thickened mucus as e.g. in cystic fibrosis.

In secretory epithelia as the intestinal mucosa, Cl^−^ secretion is mainly mediated by cystic fibrosis transmembrane conductance regulator (CFTR) which is regulated by intracellular cyclic adenosine monophosphate (cAMP). A second potential mechanism are the intracellular Ca^2+^ levels (${\text{Ca}}^{ 2+ }_{\text{i}}$) [1, 2]. Besides CFTR, Ca^2+^-activated Cl^−^ channels (CaCC) are a further mechanism for Cl^−^ secretion [2]. ${\text{Ca}}^{ 2+ }_{\text{i}}$ may also enhance the driving force for chloride secretion via activating K^+^ channels [3, 4].

The polyphenol Resveratrol (RSV) is able to affect cAMP and ${\text{Ca}}^{ 2+ }_{\text{i}}$ levels. RSV elevates cAMP levels via inhibiting phosphodiesterases and stimulating adenylate cyclase [5–7]. Increased ${\text{Ca}}^{ 2+ }_{\text{i}}$ levels were described in different tissues and cell models (e.g. vascular smooth muscle cells [8], mesothelioma cell lines [9], primary dermal fibroblasts [10] or cortical neurons [11]) but the effects were based on different mechanisms (influx from intra- or extracellular stores [8, 9], efflux inhibition [10], intracellular signalling [11]).

The effects of RSV on Cl^−^ secretion have been investigated with regard to improve the function of CFTR or the deltaF508 mutation as involved in cystic fibrosis. Activating effects were e.g. described for sinonasal epithelial cells [12, 13], a pancreatic cell line [14] and rat colonocytes [15]. Other studies failed to demonstrate effects which is discussed with regards to the models and the concentration of RSV [16, 17].

The concentration is of particular interest since the bioavailability of RSV is low so that most cells within an organism are most likely not exposed to concentrations exceeding the low micro molar range [18]. The only organ that may be exposed to higher concentrations is the small intestine due to high RSV contents in dietary supplements. Regarding the small intestine less is known about effects of RSV on Cl^−^ secretion.

Effects of RSV on intestinal cAMP-mediated Cl^−^ secretion have been described by Blumenstein et al. [19] for mouse jejunum and the epithelial cell line T84. By applying the Ussing chamber technique it could be demonstrated that RSV increased the I_sc_ (a measure of electrogenic ion transport) only in the presence of Cl^−^ [19]. RSV dimers were found to decrease CaCC-mediated currents in the epithelial cell line HT-29 and in the murine colon [20]. To our knowledge, there is only one publication that showed an effect of RSV on Ca^2+^-induced Cl^−^ secretion [21].

In a recent study [22], we investigated the effects of RSV on intestinal transport using porcine jejunal and ileal samples in Ussing chamber experiments. After incubation with RSV, porcine jejunal tissues showed a decreased I_sc_ while the ileum showed an increased I_sc_. This indicates differences between the inducibility of Cl^−^ secretion by RSV between the segments but it was not possible to evaluate whether these effects were mediated by Cl^−^ secretion. Interestingly, the increased I_sc_ occurred not in the jejunum as observed by Blumenstein et al. but in the ileum. In the study of Blumenstein et al. [19] forskolin (activator of cAMP-mediated Cl^−^ secretion), further increased the I_sc_ after RSV stimulation of jejunal tissues while we failed to induce a further increase in ileal tissues.

Based on these results the present study aimed at verifying that the RSV-mediated increase in I_sc_ in ileal tissue was due to Cl^−^ secretion. Additionally we aimed at getting first indications whether there is an effect of RSV on Ca^2+^-induced Cl^−^ secretion by measuring the effects of carbachol, which increases ${\text{Ca}}^{ 2+ }_{\text{i}}$, after incubation with RSV.

## Main text

### Materials and methods

#### Animals and tissue removal

Thirteen piglets (Sus scrofa domestica, German Landrace × Large White) kept on a conventional diet with free access to water were used. Four animals were used for the preliminary set of experiments and nine animals for the main experiments. The pigs were slaughtered by stunning with subsequent carotid artery bleeding. Tissues were removed, rinsed with cold saline (4 °C) and stored in serosal buffer (Additional file 1: Table S1) for Ussing chamber experiments.

#### Ussing chamber experiments

**Fig. 1:**
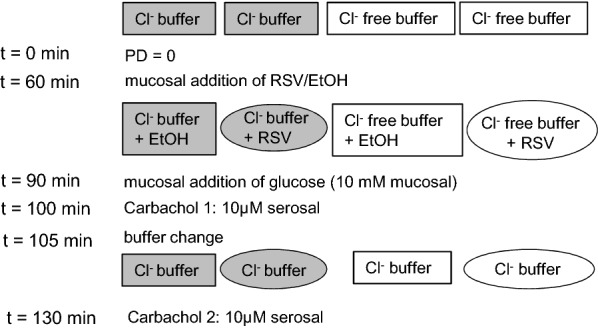
Schematic illustration of the experimental setup for Ussing chamber experiments. The composition of the serosal and mucosal buffer solutions with or without chloride is given in Additional file 1: Table S1. After an equilibration period with chloride-containing or chloride-free buffer solutions, RSV (Sigma-Aldrich, 300 µM) or ethanol (control, 20 µl/10 ml) were added to the mucosal compartment. After 30 min, glucose (10 mM mucosal) was applied in order to induce Na^+^-coupled glucose transport which is known to be inhibited by RSV [22, 25]. After another 10 min, carbachol (10 µM serosal, Sigma-Aldrich) was added to stimulate Ca^2+^-induced Cl^−^ secretion (ΔI_sc_ carbachol 1) [3]. The buffers were changed to chloride-containing buffers in all chambers and carbachol was added once again (ΔI_sc_ carbachol 2). Mucosal additions exceeding 1 mM were osmotically balanced by the serosal addition of mannitol (non-absorbable, non metabolisable sugar)

The Ussing chamber technique [23] is a in vitro setup for measuring transport processes across intact epithelia. The mucosal and serosal compartments are filled with different buffers (Fig. 1, Additional file 1: Table S1). The movement of ions across a membrane produces a potential difference. Under the applied short circuit conditions, the transepithelial potential difference (PD) is set to 0 mV using a voltage clamp devise (EC-285, Warner Instruments). The current that is necessary for setting PD to 0 mV is called the short circuit current (I_sc_) and is a measure for the transepithelial net ion transfer.

Jejunal (third meter distal to the pylorus) and ileal (first meter proximal to the ileocaecal valve, first 30 cm discarded) samples were mounted in Ussing chambers (four chambers/animal, serosal area: 1 cm^2^). The tissue conductance (G_t_) was assessed by stimulations (0.1 pps, 500 ms, 150 mV, ten times, Stimulator S48, Grass Technologies) at the beginning of the experiments and between all additions. Figure 1 explains the detailed experiment setup.

In a so far unpublished preliminary set of experiments using jejunal and ileal samples from four animals, it was tested whether the carbachol-induced ΔI_sc_ is modulated by RSV. No effect was observed for jejunal tissues (ΔI_sc_ carbachol (µA∙cm^−2^): crtl: 24.27 ± 11.52, RSV: 20.88 ± 6.62) while ΔI_sc_ carbachol for ileal tissues was decreased (crtl: 31.15 ± 4.09, RSV: 20.53 ± 4.49, p = 0.0237). Therefore, ileal tissues were used in the main experiments.

#### Data analysis and statistics

ΔI_sc_ was calculated as the difference of the I_sc_ before an addition and the maximal I_sc_ afterwards. Gaussian distribution was tested (Shapiro–Wilk normality test). In case of Gaussian distribution, RM one-way ANOVA and Fisher’s LSD test were used. Otherwise, Friedman test and uncorrected Dunn’s test were used. All these analyses were done with GraphPad Prism 7.04. The power for the RM one-way ANOVA was estimated a priori to be 0.71 (n = 8 animals) using G*Power 3 [24]. When RM one-way ANOVA was used, the post hoc calculated power (n = 9) is given in the legend to Fig. 3. Due to the high effect size, the actual power of the parametric procedure was higher as calculated a priori what indicates sufficient power for the nonparametric procedures.

### Results and discussion

**Fig. 2:**
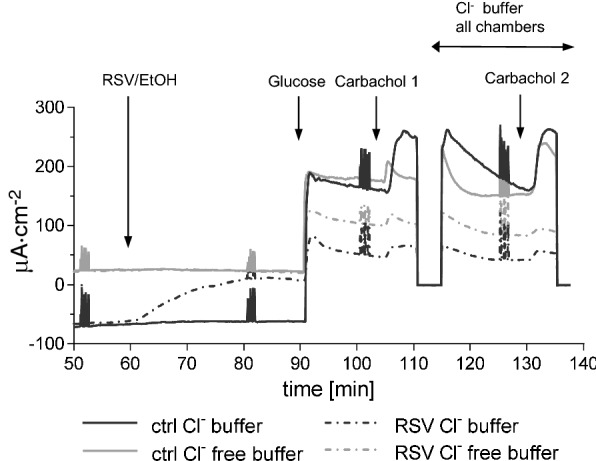
Exemplary time course of short circuit currents (I_sc_) as measured in Ussing chamber studies. The respective chambers were started with chloride containing or chloride free buffer solutions (Additional file 1: Table S1) as indicated. After the addition of RSV (300 µM mucosal), glucose (10 mM mucosal) and carbachol (10 µM serosal) the buffer solutions were changed to chloride containing buffer solutions in all chambers and carbachol was added again. The spikes in between are due to the tissue stimulation for the determination of the tissue conductance (G_t_)

**Fig. 3:**
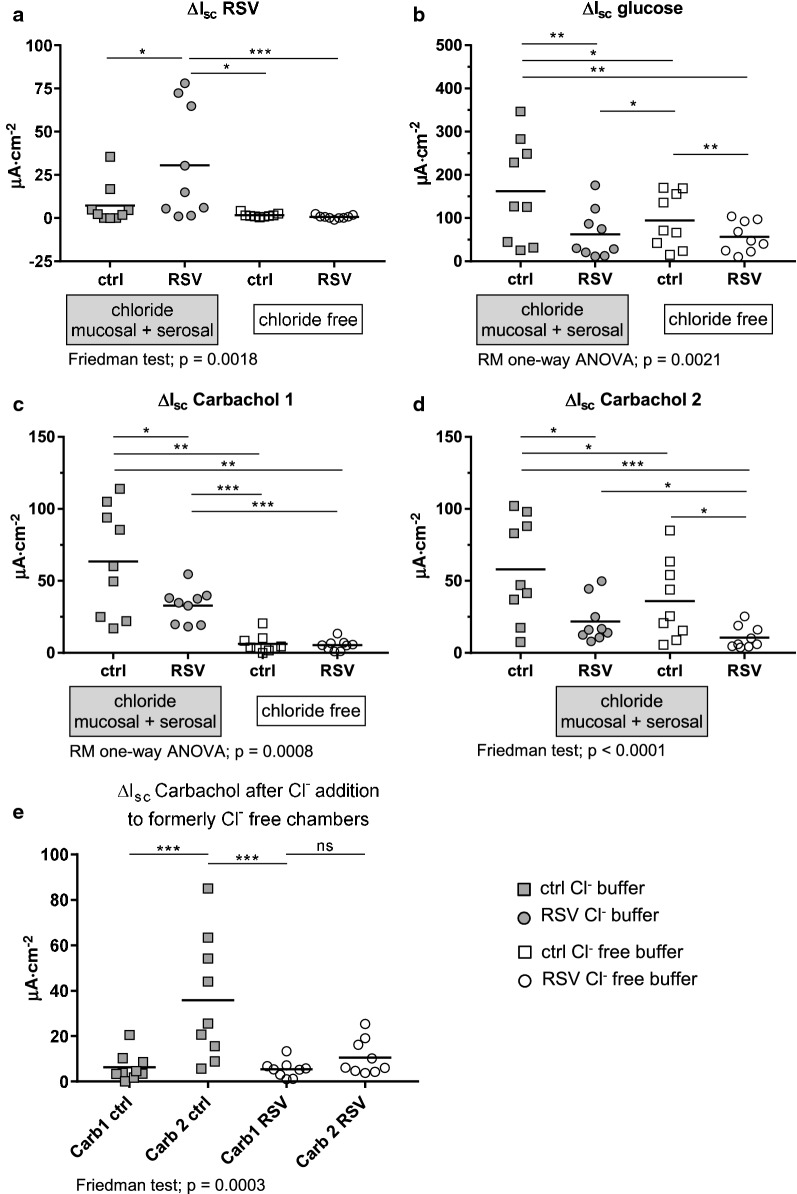
Changes in short circuit currents (ΔI_sc_, µA∙cm^−2^) as measured in Ussing chamber experiments using porcine ileal tissues and chloride containing and chloride free buffer solutions in the mucosal and serosal compartments. **a** ΔI_sc_ after the addition of Resveratrol (RSV, 300 µM mucosal). **b** ΔI_sc_ after the addition of glucose (10 mM mucosal, 10 mM mannit serosal). **c** ΔI_sc_ after the first addition of carbachol (10 µM, serosal). **d** ΔI_sc_ after the second addition of carbachol (10 µM, serosal) after the chloride free buffer solutions were replaced by chloride containing buffer solutions. **e** direct comparison of ΔI_sc_ caused by carbachol under chloride free conditions and after changing the buffers to chloride containing standard buffers. Statistic results of the respective analysis of variance are shown below the graph and the results of the post test (Fisher’s LSD after RM one-way ANOVA and uncorrected Dunn’s test after the Friedman test) are indicated with asterisks: *p ≤ 0.05, **p ≤ 0.01, ***p ≤ 0.001. For the parametric test procedures in **b** and **c**, the statistical power was calculated **b**: Power of 0.89 (η^2^ = 0.26, effect size for treatment f = 0.59); **c**: Power of 0.99 (η^2^ = 0.61, f = 1.26). Mean ± SD are given in Additional file 2: Table S2

Figure 2 shows an exemplary course of the I_sc_. Means and the statistical analysis are shown in Fig. 3. G_t_ (in mS cm^−2^) was not changed after most additions. Differences could only be observed between chambers with Cl^−^ and Cl^−^-free buffers (Cl^−^: 19.4 ± 2.18, Cl^−^ free: 14.8 ± 1.26, p = 0.0007). Glucose caused a slight increase in G_t_ in Cl^−^-containing chambers irrespective of RSV (ctrl/Cl^−^: 2.89 ± 2.53, RSV/Cl^−^: 3.02 ± 2.95).

As shown in Fig. 3a, I_sc_ was increased by RSV (p = 0.029) what validates the results from our previous study [22]. Since this increase was absent under Cl^−^-free conditions (p = 0.0001), this part of the study confirms, that the RSV-mediated increase is caused by Cl^−^ secretion as it was speculated.

As shown in Fig. 3b, RSV decreased the glucose-induced ΔI_sc_ (p = 0.002), what is already known from our previous studies [22, 25]. Additionally, this part of the study may give some new indications that RSV may affect Ca^2+^-induced Cl^−^ secretion since the glucose-induced I_sc_ for ctrl/Cl^−^-free chambers was decreased compared to ctrl/Cl^−^ chambers (p = 0.014). It has been shown that glucose stimulates Ca^2+^-induced Cl^−^ secretion in intestinal cells [26, 27]. Therefore, this difference may indicate that a part of the ΔI_sc_ under control conditions may be due to glucose-mediated stimulation of Ca^2+^-induced Cl^−^ secretion especially since there is a correlation between the glucose-induced ΔI_sc_ under ctrl and Cl^−^-free conditions (R^2^ = 0.846, p = 0.0004). Nevertheless, there is still a difference between the glucose-induced ΔI_sc_ in Cl^−^-free control chambers and RSV/Cl^−^-free chambers (p = 0.0066), while the ΔI_sc_ for RSV-treated tissues does not depend on the presence of Cl^−^ (p = 0.577).

Taken together, the results in Fig. 3b confirm the inhibition of Na^+^-coupled glucose transport by RSV and point to an inhibitory potential with regard to glucose-stimulated Ca^2+^-induced Cl^−^ secretion.

This is strengthened by the data in Fig. 3 c, d and e. In the presence of Cl^−^, the carbachol-induced ΔI_sc_ was decreased after RSV treatment (p = 0.034). Without Cl^−^, a response to carbachol was observed neither in control nor in RSV-treated chambers. The difference between the RSV-treated chambers with and without Cl^−^ (p = 0.0004) may indicate an incomplete inhibition of Ca^2+^-dependent Cl^−^ secretion. After the buffer solutions were changed to chloride-containing buffer in all chambers and carbachol was added again (ΔI_sc_ carbachol 2, Fig. 3d) the formerly Cl^−^-free chambers responded in a similar way as the Cl^−^-containing chambers. The responses in the chambers with Cl^−^ during the whole experiment were still higher than in the former Cl^−^-free chambers but the differences were less pronounced compared to ΔI_sc_ carbachol 1. Figure 3e also clearly shows, that the response to carbachol is restored in control chambers after the readdition of Cl^−^ (p = 0.0003). This is not the case for RSV-treated chambers (p = 0.1441).

In summary, the results of the present study demonstrate that RSV (1) induces chloride secretion and (2) inhibits Ca^2+^-induced Cl^−^ secretion in the porcine ileum.

This raises the question what the basis for the RSV-induced Cl^−^ secretion is. This could not finally be elucidated from the present data but it seems reasonable to assume that a cAMP-mediated activation of CFTR may be the reason for the RSV-induced Cl^−^ secretion as it was the case in the murine jejunum [19]. If the observed Cl^−^ secretion would be due to CFTR activation, this might explain why RSV had no effect on ileal forskolin-induced I_sc_ in our previous study [22], when assuming that CFTR is working at its maximal capacity after activation by RSV.

The inhibitory effect of RSV on Ca^2+^-induced Cl^−^ secretion is, with regard to the ability of RSV to increase ${\text{Ca}}^{ 2+ }_{\text{i}}$, a surprising finding but to our knowledge, ${\text{Ca}}^{ 2+ }_{\text{i}}$ after short time exposure to RSV has never been measured in enterocytes. It has to be questioned, at which stage RSV affects the action of carbachol. Firstly, RSV may not lead to increased ${\text{Ca}}^{ 2+ }_{\text{i}}$ but rather inhibit the carbachol-induced increase in ${\text{Ca}}^{ 2+ }_{\text{i}}$ since it has been shown in Caco-2 cells, that RSV prevents Ca^2+^ mobilization from the endoplasmic reticulum that was induced by the non-steroidal anti-inflammatory drug indomethacin [28]. Secondly, inhibitory effects on K^+^ channels may be discussed but assuming this, the RSV-evoked basal Cl^−^ secretion is difficult to explain. Also direct RSV-transporter interactions may be involved as discussed for the activating effects on the CaCC TMEM16A [21].

In conclusion, it has to be noted that the effects of RSV on intestinal chloride secretion are different between the proximal and distal parts of the small intestines. In addition, both cAMP- and Ca^2+^-mediated chloride secretion is involved and is affected differently. These complex effects should be subjected to further studies since they may contribute to develop a concept about the variety of effects that RSV exerts in different organs or cell culture models. In any case, it becomes increasingly clear, that it is difficult to generalize effects of RSV between intestinal locations, organs, cell culture models or species.

## Limitations

The Ussing chamber technique as applied in the present study only gives information about changes in the transepithelial net ion transfer. Under the experimental conditions it is not possible to distinguish between the movements of different ions. Therefore, it is not possible to evaluate whether Ca^2+^ is still able to the cell after incubation with RSV or whether changes in the K^+^ conductance of the membrane are involved in the observed effects. This limits the significance of the study with regard to mechanistic aspects.

## Additional files

**Additional file 1.** Composition of buffer solutions for Ussing chamber experiments.

**Additional file 2.** Means ± standard deviation for the data shown in Fig. 3 as dot plots with means only.

## Abbreviations

Ca^2+^: calcium
Ca_i_^2+^: intracellular calcium concentration
CaCC: Ca^2+^ activated Cl^−^ channel
cAMP: cyclic adenosine monophosphate
CFTR: cystic fibrosis transmembrane conductance regulator
Cl^−^: chloride
G_t_: tissue conductance
I_sc_: short circuit current
K^+^: potassium
RSV: Resveratrol
