# Supplementary material for: Calcium-induced chloride secretion is decreased by Resveratrol in ileal porcine tissue
Source: BMC Res Notes. 2018 Oct 11;11:719. doi: 10.1186/s13104-018-3825-4 (PMC6182809; doi:10.1186/s13104-018-3825-4)
Supplement: Supplementary file 1 — Additional file 1. Composition of buffer solutions for Ussing chamber experiments. [file 13104_2018_3825_MOESM1_ESM.pdf]

Table S1 Composition of buffer solutions for Ussing chamber experiments

Cl^- containing buffer solution		Cl^- free buffer solutions	
	mM		mM
NaCl	117.0	$C_6H_{11}NaO_7$	117.0
KCl	4.7	$C_6H_{11}KO_7$	4.7
$CaCl_2 \cdot 2\ H_2O$	2.5	$(C_6H_{11}O_7)_2Ca \cdot H_2O$	2.5
$MgSO_4 \cdot 7\ H_2O$	1.2	$MgSO_4 \cdot 7\ H_2O$	1.2
NaH_2PO_4	1.2	NaH_2PO_4	1.2
$NaHCO_3$	25.0	$NaHCO_3$	25.0
additional components in the serosal and mucosal buffer solutions			
Glucose (serosal)	10.0		
Mannitol (mucosal)	10.0		

All buffer solutions were kept at 39 °C, and were continuously aerated with carbogen (final pH 7.42). To create Cl^- free conditions, Cl^- was substituted by gluconate. Serosal buffers contained 10 mM glucose that was replaced by 10mM mannitol in the mucosal buffer solutions. All chemicals were ordered from Sigma-Aldrich, St. Louis, USA expect NaCl and KCl (Carl Roth GmbH + Co.KG, Karlsruhe, Germany), $(C_6H_{11}O_7)_2Ca \cdot H_2O$ (Fluka, Buchs Switzerland, now Sigma-Aldrich, St. Louis, MO, USA) and glucose (Merck KGaA, Darmstadt, Germany).
