# Supplementary material for: Calcium-induced chloride secretion is decreased by Resveratrol in ileal porcine tissue
Source: BMC Res Notes. 2018 Oct 11;11:719. doi: 10.1186/s13104-018-3825-4 (PMC6182809; doi:10.1186/s13104-018-3825-4)
Supplement: Supplementary file 2 — Additional file 2. Means ± standard deviation for the data shown in Fig. 3 as dot plots with means only. [file 13104_2018_3825_MOESM2_ESM.pdf]

Table S2 Means ± standard deviation for the data shown in figure 3 as dot plots with means only

**Fig. 3a: ΔI_{sc} RSV**	**$\mu A \cdot cm^{-2}$**	**Fig. 3b: ΔI_{sc} glucose**	**$\mu A \cdot cm^{-2}$**
ctrl/Cl^-	7.29 ± 11.81	ctrl/Cl^-	162.6 ± 118.7
RSV/Cl^-	30.49 ± 32.36	RSV/Cl^-	62.67 ± 57.18
ctrl/Cl^- free	1.63 ± 1.10	ctrl/Cl^- free	94.46 ± 63.36
RSV/Cl^- free	0.68 ± 0.99	RSV/Cl^- free	56.33 ± 35.44
**Fig. 3c: ΔI_{sc} Carbachol 1**	**$\mu A \cdot cm^{-2}$**	**Fig. 3d: ΔI_{sc} Carbachol 2**	**$\mu A \cdot cm^{-2}$**
ctrl/Cl^-	63.62 ± 37.48	ctrl/Cl^-	58.03 ± 35.45
RSV/Cl^-	32.86 ± 11.97	RSV/Cl^-	21.96 ± 15.13
ctrl/Cl^- free	6.29 ± 6.19	ctrl/Cl^- free	35.89 ± 27.29
RSV/Cl^- free	5.43 ± 3.74	RSV/Cl^- free	10.64 ± 7.79
